# Design, Implementation, and Evaluation of a Multidisciplinary Palliative Care Curriculum for Obstetrician Gynecologist Residents

**DOI:** 10.1089/pmr.2024.0082

**Published:** 2025-02-28

**Authors:** Lindsay W. Brubaker, Marisa R. Moroney, Aneesa Thannickal, Meredith J. Alston, Josephine H. Amory, Amanika Kumar, Carolyn Lefkowits

**Affiliations:** ^1^ Department of Obstetrics and Gynecology, Division of Gynecologic Oncology, University of Colorado, Aurora, Colorado, USA.; ^2^ Department of Obstetrics and Gynecology, Mayo Clinic, Rochester, Minnesota, USA.; ^3^ Department of Obstetrics and Gynecology and Denver Health Medical Center, University of Colorado, Aurora, Colorado, USA.; ^4^ Division of Palliative Care, Department of Family Medicine, University of Washington, Seattle, Washington, USA.; ^5^ Division of Gynecologic Oncology, Department of Obstetrics and Gynecology, Mayo Clinic, Rochester, Minnesota, USA.

**Keywords:** OBGYN, palliative care, residency curriculum

## Abstract

**Abstract:**

Palliative care focuses on providing relief from the stress and symptoms of serious illness. Obstetrician gynecologists (OBGYNs) manage a variety of complex clinical situations ranging from delivering a cancer diagnosis to caring for a neonatal demise to symptom management, all of which fall within the realm of palliative care. Palliative care is relevant to the practice of OBGYNs, yet residents rarely receive formal primary palliative care training. We sought to design, implement, and evaluate a dedicated curriculum on palliative care for OBGYN residents.

**Abstract:**

We performed a needs assessment of an OBGYN residency at a single institution. This information was used to develop a multidisciplinary palliative care curriculum. Post-curriculum surveys were distributed and analyzed. Descriptive statistics were utilized.

**Abstract:**

Our needs assessment identified a lack of knowledge and competency in palliative care concepts and skills. Using this information, we created a multidisciplinary palliative care curriculum, including clinical experiences, chalk talks, and online didactics. Post-curriculum surveys revealed a marked improvement in both knowledge base and perceived competence. Feedback was overwhelmingly positive; residents indicated that the curriculum was both a valuable use of time and would impact their provision of care in the future.

**Abstract:**

A dedicated palliative care curriculum is an impactful addition to OBGYN training.

## Introduction

Palliative care is defined by the Center to Advance Palliative Care as:

*specialized medical care for people living with a serious illness. This type of care is focused on providing relief from the symptoms and stress of a serious illness. The goal is to improve quality of life for both the patient and the family. Palliative care is provided by a specially-trained team of doctors, nurses and other specialists who work together with a patient’s other doctors to provide an extra layer of support. Palliative care is based on the needs of the patient, not on the patient’s prognosis. This care is appropriate at any age and at any stage in a serious illness.^1^*

Palliative care includes symptom management, communication and shared decision-making, spiritual and psychosocial assessment, and advanced care planning. Palliative care also includes, but is not limited to, hospice and end-of-life care. The provision of palliative care has been demonstrated in multiple studies to convey clinical benefits both for the patient and their families, including improved symptom control and satisfaction with care.^2^ While the positive impact of palliative care is clear, the supply of specialty palliative care clinicians is not sufficient enough to provide palliative care to all who might benefit.^3^ As a result, there is a focus on primary palliative care. Primary palliative care refers to palliative care of the patient’s serious illness delivered by the patient’s primary health care provider or team; this is distinct from specialty palliative care, which is delivered by a team of palliative care specialists.^4,5^ The Institute of Medicine has declared that “all clinicians across disciplines and specialties who care for people with advanced serious illness should be competent in basic palliative care, including communication skills, interprofessional collaboration, and symptom management… Educational institutions and professional societies should provide training in palliative care domains throughout the professional’s career.”^6^ To provide comprehensive women’s health care, obstetrician gynecologists (OBGYNs) must be equipped to function as primary palliative care providers.

Palliative care is highly relevant to the practice of obstetrics and gynecology. OBGYNs treat difficult symptoms, including pain and nausea, which require an in-depth understanding of symptom management options. Gynecologists routinely deliver serious news, including a new cancer diagnosis, early pregnancy loss, or the diagnosis of chronic or limiting diseases such as primary ovarian failure or endometriosis. Similarly, obstetricians diagnose fetal malformations and stillbirths and care for women through life-threatening pregnancies. All of these difficult conversations are best handled when providers use high-level communication skills, skills that can be learned through the lens of palliative care.

Perinatal palliative care, a subset of palliative care particularly relevant to OBGYN, is developing into a field of its own. Perinatal palliative care is focused on the care of the mother-baby unit when the fetus is diagnosed with a life-threatening or life-limiting condition, including extreme prematurity.^7^ Although the symptom management of the infant is often managed by the neonatal team, obstetricians are responsible for the prenatal counseling and shared decision-making with the mother and family.^4^ In these scenarios, specialists recommend the use of an Advanced Care Birth Plan, conceptually similar to advanced care plans used in adult palliative care.^8^ Through the use of an Advanced Care Birth Plan, families are able to share their hopes, concerns, and what is most important to them with their perinatal care providers. These goals of care are translated into a delivery and neonatal intervention plan, which will remain the guiding principle, though the specifics may shift as the clinical situation evolves after delivery. As with all aspects of palliative care, the intention of the Advanced Care Birth Plan is to improve a family’s sense of control and understanding of their situation and thus to allow for shared decision-making.^8^

Despite the profound relevance of palliative care to obstetrics and gynecology, OBGYN residents typically receive little formal education in palliative care skills; specifically, sharing serious news, exploring goals of care, or practicing shared decision-making, as well as complex symptom management. These skills have been deemed critical to our training and are incorporated into the ACGME milestone evaluation framework, specifically the professionalism and interpersonal and communication skills milestones. However, there is no formal national curriculum available to reinforce these concepts, nor is there any existing published data on curricular efforts. Our aim was to design, implement, and evaluate a multidisciplinary palliative care curriculum for OBGYN residents.

## Methods

### Needs assessment

A needs assessment was developed using validated questions from existing palliative care educational surveys.^9–11^ Specifically, competency questions were derived from a gynecological oncology fellow needs assessment, knowledge-based questions obtained from the *Palliative Care Knowledge Examination* written by Weissman et al., and questions about degree of experience from a published, resident-led palliative care education project.^9–11^ “Palliative care” and “formal training” were explicitly defined within the needs assessment tool. The needs assessment began with questions related to the residents’ perceived competency compared with the competency of a “skilled attending obstetrician-gynecologist.” These questions were asked based on a Likert scale of 1 to 5, with 1 being “not competent” and 5 being “competent,” and addressed skills related to communication skills and symptom management. The residents were then asked to rank their top three preferred methods of learning palliative care content. We included a series of multiple-choice questions to test knowledge on basic concepts of palliative care and symptom management, particularly those that may be relevant to OBGYN care. Residents were also queried regarding their previous palliative care training and their perception of the role of palliative care in the field of obstetrics and gynecology. Prior to distribution, the survey tool was piloted with current fellows within the same OBGYN department for readability and clarity of questions. Surveys were anonymous. Each participant was asked to create a unique code to allow linking of pre- and postresults. A complete needs assessment is available online.

The needs assessment was delivered electronically to all active residents at a single academic medical center (*n* = 41). Three reminder emails were sent. A waiver was included with the introductory email that explained the intention and voluntary nature of the survey. The study was reviewed and considered exempt from formal review by the Colorado Institutional Review Board. The needs assessment was distributed to the entire resident cohort over two academic years, reaching a total of 41 residents at a single institution, with a response rate of 100%.

### Curriculum development and implementation

**FIG. 1.**
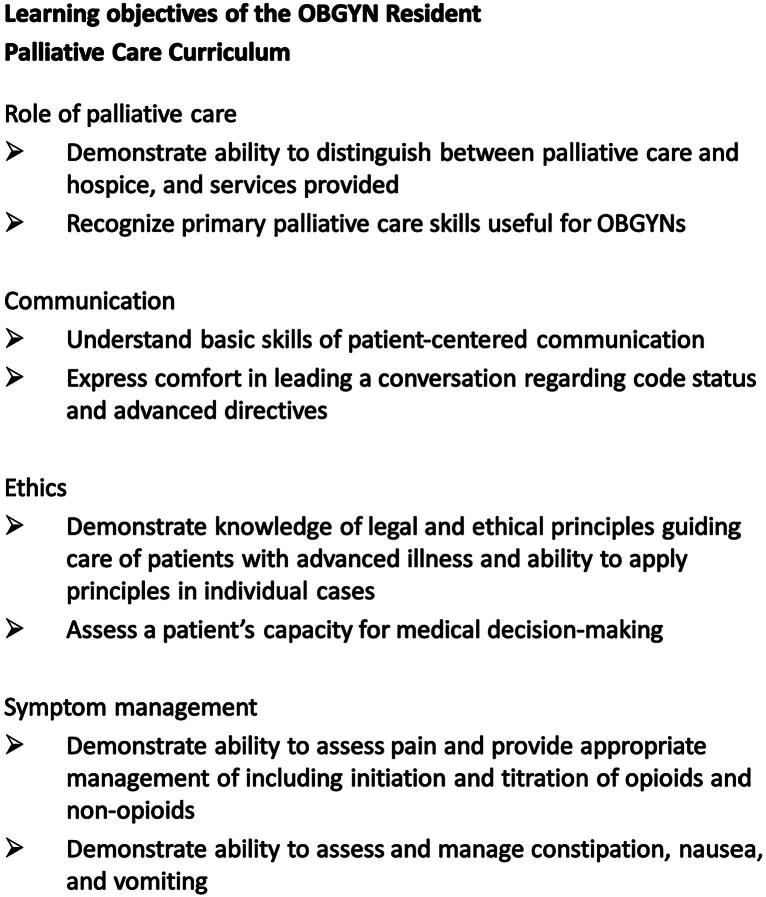
Learning Objectives of OBGYN Palliative Care Curriculum.

**FIG. 2.**
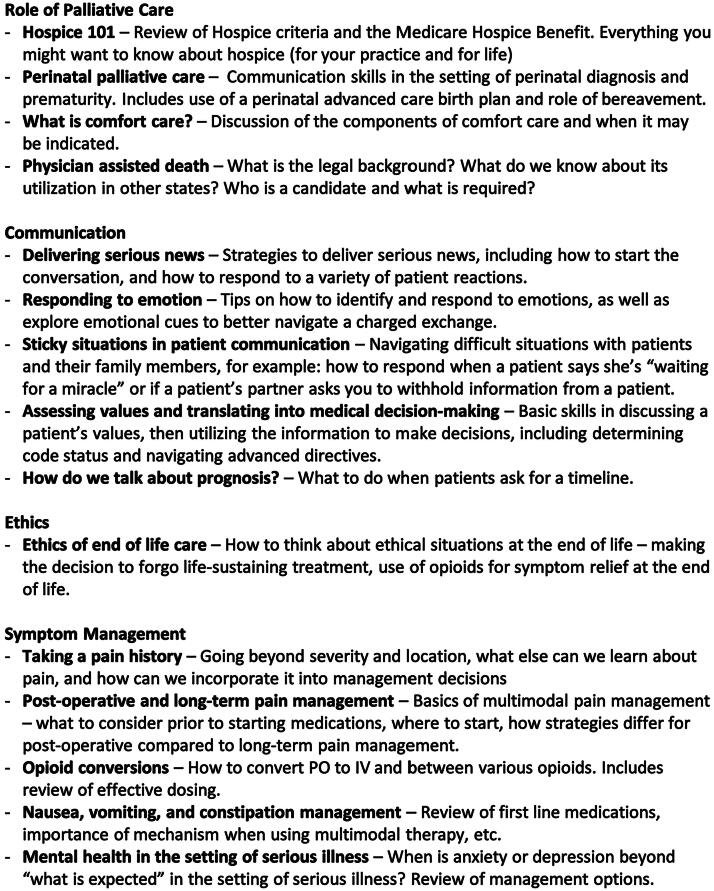
Chalk Talk Topics.

Using the results of our needs assessment, we worked with palliative care and perinatal palliative care specialists to create a curriculum for mid-level OBGYN residents. We utilized the ACGME milestones, as well as the domains of palliative care from the National Consensus Project for Quality Palliative Care, to serve as a backbone for the objectives of the rotation (Fig. 1).^12^ Working within an existing subspecialty outpatient rotation, we protected a total of four half-days for palliative care training, including clinical experiences, chalk talks, and online didactics. The clinical experiences were inpatient palliative care, outpatient palliative care, and home/inpatient hospice. There were two required chalk talks: advanced directives and hospice. Residents were provided with a list of “chalk talk” topics and were asked to select three additional topics of greatest interest to them (Fig. 2). Chalk talks were each 10 minutes long, conducted by the faculty medical director for the curriculum who is fellowship-trained in palliative care. Residents were also required to review an online presentation on perinatal palliative care with voiceover from a perinatal palliative care specialist. Finally, residents were asked to complete a series of online modules available through the Center to Advance Palliative Care (CAPC; www.capc.org), an online member-based organization comprised of hospitals, academic centers, hospices, and other organizations.^13^ Out of over 40 modules available, the creators of the curriculum reviewed the content to identify those that would be most relevant and most impactful through online delivery, including pain and symptom management. Seven modules were selected, including opioid prescribing, side effects, and conversions, as well as nausea and vomiting, depression, and anxiety. We estimated each module would take 15–30 minutes. Resident trainees were given time within the work week for this self-study. The residents were also provided a list of additional resources, including relevant literature and the website for VitalTalk (www.vitaltalk.org), an online communication resource.^14^

After completing the rotation, each resident met for 10 minutes with the faculty curriculum medical director to debrief their experience. They were asked to complete a voluntary post-curriculum survey, which was distributed electronically the weekend after the end of the rotation and queried the same competencies and knowledge-based questions as the needs assessment. At the conclusion of the first academic year, we analyzed the post-curriculum surveys from the residents who participated in the OBGYN Resident Palliative Care Curriculum and completed the post-curriculum survey.

## Results

### Needs assessment

**FIG. 3.**
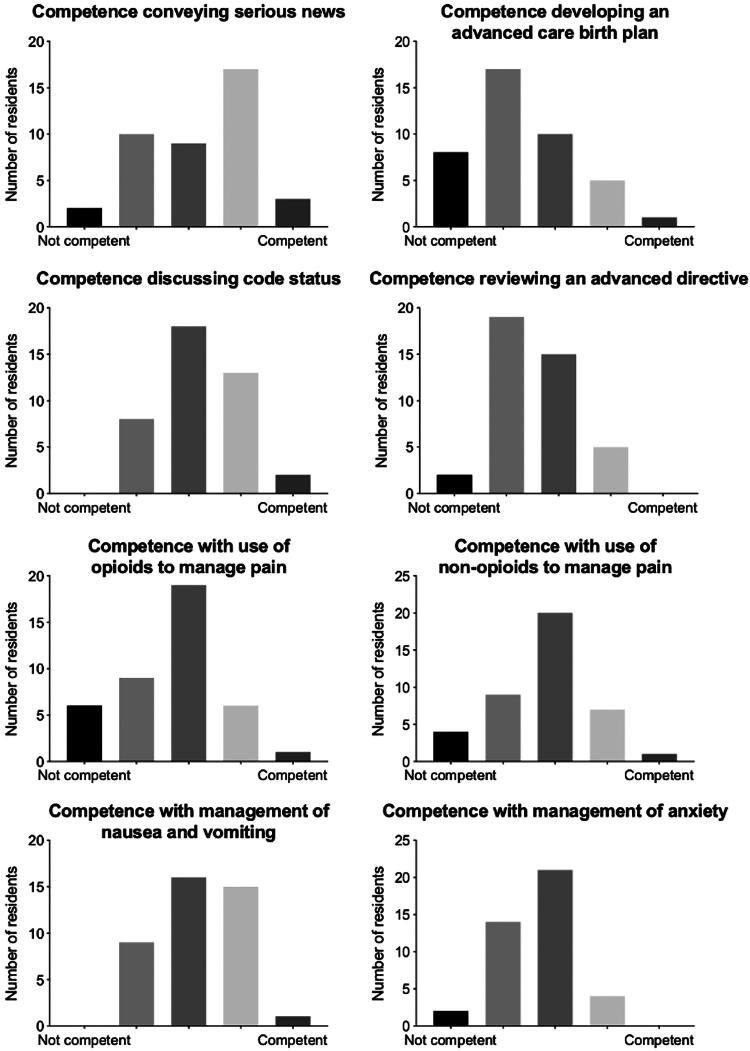
Pre-curriculum OBGYN resident self-reported competence in palliative care skills.

Ten percent or less of all residents included in the pre-curriculum needs assessment (*n* = 41) described themselves as competent with any palliative care skill (Fig. 3), including reviewing advanced directives or determining decision-making capacity. Notably, in regard to competency in the use of opioids to manage pain, 15% of residents described themselves as “not competent.” For the knowledge-based questions, only one in three residents answered each question correctly on average, with a range of 17–68% (Table 1).

**Table 1.** Percentage of Knowledge-Based Questions Answered Correctly by OBGYN Residents during the Needs Assessment (*n* = 41)

Multiple choice question	Answered correctly	
	%	*N*
Equivalence between PO and IV morphine	17%	7
Division of short and long-acting opioid over 24 hours	20%	8
Calculation for breakthrough pain medication dosing	34%	14
Timing of maximal analgesic effect after Percocet	39%	16
Counseling regarding nausea after morphine	56%	23
Transmitters mediating the chemoreceptor trigger zone	27%	11
Least appropriate as needed medication for constipation	27%	11
Criteria for decision making capacity	34%	14
Role of functional ability in prognosis	68%	28
Role of intravenous fluids in end-of-life care	49%	20
Medicare hospice benefit criteria	24%	10

When asked whether palliative care was relevant to OBGYN practice, 95% stated they either agreed or strongly agreed. Similarly, when asked whether OBGYN residents should undergo training in palliative care, 93% stated they either agreed or strongly agreed. When asked to rank preferences regarding a variety of teaching methods, clinical experiences, informal chalk talks, and lecture-based didactics were ranked highest.

### Post-curriculum assessment

Our post-curriculum survey response rate was 88% with responses from eight of the nine residents who participated in the curriculum. Broadly, we found notable improvement across the competencies. When comparing the eight residents to their own matched pre-curriculum needs assessments, over 50% of residents demonstrated improvement in all 17 competencies. On average, each of the eight respondents reported an improvement in 76% (13/17) of the addressed competencies. The most marked progress was noted in goals of care, advanced directives, and communication with patients regarding denial of disease seriousness. We also saw progress in the assessment and management of pain, nausea/vomiting, anxiety, and depression—in these competencies, all or all but one of the respondents demonstrated improvement.

Even at the completion of the curriculum, none of the respondents described themselves as “competent” (top of the Likert scale). This may be explained by the fact that more in-depth exposure to the field may have generated increased awareness about the breadth of knowledge needed for true “competency.” Another consideration is that the residents participating in this course were post-graduate year two and three residents and were asked to compare their competency to that of an attending physician, which midlevel residents may have viewed as an unattainable level of competence.

Upon review of the knowledge-based questions, the majority either maintained the correct answer or newly selected the correct answer. Specifically, the average percentage of correct answers improved from 32% to 66% (proportions Z score 4.52, *p* < 0.00001). When considering only the questions addressing pain management and narcotic use, the percentage answered correctly improved from 33% to 73% (proportions Z score 3.58, *p* = 0.00017).

When asked about the preferred method of learning after having participated in the curriculum, clinical experiences and chalk talks were unanimously ranked as the top modalities. Residents also unanimously reported that of the clinical experiences, inpatient palliative care was most impactful.

Finally, we asked global questions to understand the potential impact of the curriculum. When asked whether palliative care is relevant to OBGYN practice, all the respondents who completed the curriculum and participated in the post-curriculum survey stated that they agreed or strongly agreed. This was consistent with the pre-curriculum finding of 95% of residents who stated palliative care is relevant to OBGYN practice. Similarly, all but one resident (87.5%, 7 of 8 respondents) stated they agreed or strongly agreed that OBGYNs should undergo training in palliative care. When asked about the value of the curriculum—understanding the time limitations in clinical training—all respondents answered with affirmation that the material was relevant and worth the time spent. Five of the eight residents responded with “the material is relevant, and it was the perfect amount of time” while the remaining three responded with “the material is relevant, but it requires more time.”

When asked in an open-ended fashion, “what aspect of this curriculum will most impact your future practice?” the following concepts were commonly addressed:

- Communication with patients and families and learning to deliver bad news
- Opioid dosing and conversions
- Experience in holding difficult family meetings
- Management of chronic pain

When asked what else they would like in this curriculum moving forward, the most common request was a perinatal palliative care clinical experience, as well as more inpatient palliative care exposure.

## Discussion

Palliative care has been demonstrated to improve patient and family satisfaction, as well as clinical outcomes.^2^ However, there are a limited number of palliative care providers and a critical need to increase capacity in the provision of palliative care. With proper training in palliative care skills, OBGYNs have the potential to serve as leaders in primary palliative care and as collaborators with our specialty palliative care colleagues.

In the course of this work, we have confirmed the relevance of and need for dedicated palliative care training within OBGYN residency. We have also created a curriculum that raised competency and knowledge base and was broadly accepted as worth the time and effort in a busy OBGYN residency.

The most significant challenge in our effort was to identify clinical learning experiences in perinatal palliative care. We identified this experience as important for residents given the common practice of obstetrics among OBGYNs. However, at our institution, it was difficult to plan in advance when these critical conversations with a mother and family might occur. We found multidisciplinary treatment planning meetings, attended by high-risk obstetricians, neonatologists, and fetal surgeons, to be potential opportunities for clinical learning; however, most of the treatment decision-making had already happened by the time these meetings occurred. Given our inability to identify a consistent source of clinical exposure, we utilized a presentation by a palliative care and maternal fetal medicine fellowship-trained colleague at another institution as the primary source of content for the perinatal portion of the course. Moving forward, we hope to create a presentation focused on Advanced Care Birth Plan creation and utilization until a more sustainable clinical experience can be established.

Armed with our identification of a need and an overwhelmingly positive response to our curriculum initiative, we have begun the process to expand the effort to integrate palliative care training into OBGYN residencies at other institutions. We are starting by sharing our needs assessment so that institutions may identify their own needs. Next, we hope to encourage dissemination of our curriculum and further development of additional creative, multidisciplinary approaches to training in palliative care for OBGYN residents.

## Abbreviations Used

ACGME: Accreditation Council for Graduate Medical Education
CAPC: Center to Advance Palliative Care
OBGYN: Obstetrics and Gynecology
